# Differential behavioral and physiological effects of anodal transcranial direct current stimulation in healthy adults of younger and older age

**DOI:** 10.3389/fnagi.2014.00146

**Published:** 2014-07-10

**Authors:** Kirstin-Friederike Heise, Martina Niehoff, J.-F. Feldheim, Gianpiero Liuzzi, Christian Gerloff, Friedhelm C. Hummel

**Affiliations:** ^1^Brain Imaging and Neurostimulation (BINS) Laboratory, Department of Neurology, University Medical Center Hamburg-EppendorfHamburg, Germany; ^2^Department of Neurology, University Hospital ZürichZürich, Switzerland

**Keywords:** aging, anodal tDCS, manual dexterity, short-interval intracortical inhibition, TMS

## Abstract

Changes in γ-aminobutyric acid (GABA) mediated synaptic transmission have been associated with age-related motor and cognitive functional decline. Since anodal transcranial direct current stimulation (atDCS) has been suggested to target cortical GABAergic inhibitory interneurons, its potential for the treatment of deficient inhibitory activity and functional decline is being increasingly discussed. Therefore, after-effects of a single session of atDCS on resting-state and event-related short-interval intracortical inhibition (SICI) as evaluated with double-pulse TMS and dexterous manual performance were examined using a sham-controlled cross-over design in a sample of older and younger participants. The atDCS effect on resting-state inhibition differed in direction, magnitude, and timing, i.e., late relative release of inhibition in the younger and early relative increase in inhibition in the older. More pronounced release of event-related inhibition after atDCS was exclusively seen in the older. Event-related modulation of inhibition prior to stimulation predicted the magnitude of atDCS-induced effects on resting-state inhibition. Specifically, older participants with high modulatory capacity showed a disinhibitory effect comparable to the younger. Beneficial effects on behavior were mainly seen in the older and in tasks requiring higher dexterity, no clear association with physiological changes was found. Differential effects of atDCS on SICI, discussed to reflect GABAergic inhibition at the level of the primary motor cortex, might be distinct in older and younger participants depending on the functional integrity of the underlying neural network. Older participants with preserved modulatory capacity, i.e., a physiologically “young” motor network, were more likely to show a disinhibitory effect of atDCS. These results favor individually tailored application of tDCS with respect to specific target groups.

## Introduction

Changes in the excitation-inhibition balance with advancing age involving alterations of γ-aminobutyric acid (GABA) mediated synaptic transmission (Grachev and Apkarian, 2001; Grachev et al., 2001; Pinto et al., 2010; Gaetz et al., 2011) have been proposed as (one) underlying mechanism for age-related motor and cognitive functional decline (Gleichmann et al., 2011). Regarding the direction of alterations of the human motor system however, controversial findings exist for respective surrogate markers of GABAergic inhibition extracted from various electrophysiological methods (Hortobagyi et al., 2006; Smith et al., 2009; McGinley et al., 2010; Marneweck et al., 2011; Rossiter et al., 2014). It has previously been shown that deficient resting-state motorcortical inhibition determines poor event-related modulation, i.e., less fast and precise release of inhibition during movement preparation, a finding that was closely associated with decrements in manual dexterity (Heise et al., 2013). Moreover, insufficient induction of cortical plasticity in response to motor training has also been related to imbalanced inhibitory activity in older participants (Sawaki et al., 2003; Fujiyama et al., 2009; Rogasch et al., 2009).

In times of an average life expectancy far beyond 70 years of age in high-income countries (World Health Organization, 2012), ameliorating the negative prize that comes with advancing age seems appealing. On the basis of findings in young healthy participants, it has been suggested that modulating cortical excitability with anodal transcranial direct current stimulation (atDCS) is causally linked to behavioral improvement in the cognitive and motor domain (Nitsche et al., 2003c, 2006; Antal et al., 2004a,b; Fregni et al., 2005; for review Reis and Fritsch, 2011; Sohn et al., 2012). Anodal tDCS over the primary motor cortex (MI) led to enhanced motor performance (Boggio et al., 2006; Vines et al., 2006; Cogiamanian et al., 2007; Matsuo et al., 2011) and increased efficiency of motor learning in healthy participants in a variety of behavioral paradigms (Galea and Celnik, 2009; Hunter et al., 2009; Reis et al., 2009; Stagg et al., 2011a,c).

Since mild direct current stimulation most likely affects horizontal intracortical interneurons of which GABAergic neurons represent the largest neuronal population which exert strong inhibition upon the pyramidal cell (Lang et al., 2011), GABA-mediated inhibition has been proposed to constitute one potential target of atDCS (Nitsche et al., 2004, 2005; Stagg et al., 2009, 2011b). Hence, for conditions marked by deficient inhibitory activity as it has been shown to occur on the one hand in disease, like following a stroke (Hummel et al., 2009; Edwards et al., 2013; Honaga et al., 2013; Liuzzi et al., 2014), in movement disorders (Beck et al., 2008; Heise et al., 2010; Jackson et al., 2013), or on the other hand in the course of healthy aging (McGinley et al., 2010; Marneweck et al., 2011; Heise et al., 2013), this mechanism would be of particular interest and could offer a potential tool to target age-related functional decline. In accordance with this idea, enhanced motor behavior and augmented effects of motor training have been found after stimulation in participants of older age (Hummel et al., 2010; Goodwill et al., 2013; Zimerman et al., 2013).

Therefore, one point of interest of the present work was whether atDCS is able to perturb intracortical inhibition measured by means of double-pulse transcranial magnetic stimulation (dpTMS) in resting-state and event-related conditions. The driving hypothesis was that level of resting-state inhibition is shifted as to open up a window and subsequently allow for more pronounced event-related modulation to occur. Furthermore, evidence for a direct association between stimulation induced changes in motorcortical excitability/inhibition and motor function is scarce. For that reason the present work addressed the question whether atDCS-induced changes in the intracortical inhibitory network within the primary motor cortex would be associated in direction and magnitude with stimulation-induced changes in dexterous motor behavior in older and younger participants.

## Materials and methods

### Participants

Young (*N* = 16 young average age 24.27 ± 1.6 years, range 22–28, 8 female) and old (*N* = 16 old average age 73.4 ± 6.3 years, range 65–83, 7 female) healthy participants volunteered in the experiment. All were right-handed (Oldfield, 1971) and none reported a history of serious medical, neurological or psychiatric diseases or any contraindications for tDCS or TMS, as probed by a standardized questionnaire based on available safety recommendations (Nitsche et al., 2008; Rossi et al., 2009). In all participants, the score of Mini-Mental State Examination (Folstein et al., 1975) was ≥29/30. Subjects were naïve to the experimental purpose and none of them were professional piano players or trained as a typist. All participants gave full written informed consent to participate in the experiment in accordance with the ethics committee of the Medical Counsel Hamburg (protocol number PV3770).

### Experimental set up

**Figure 1:**
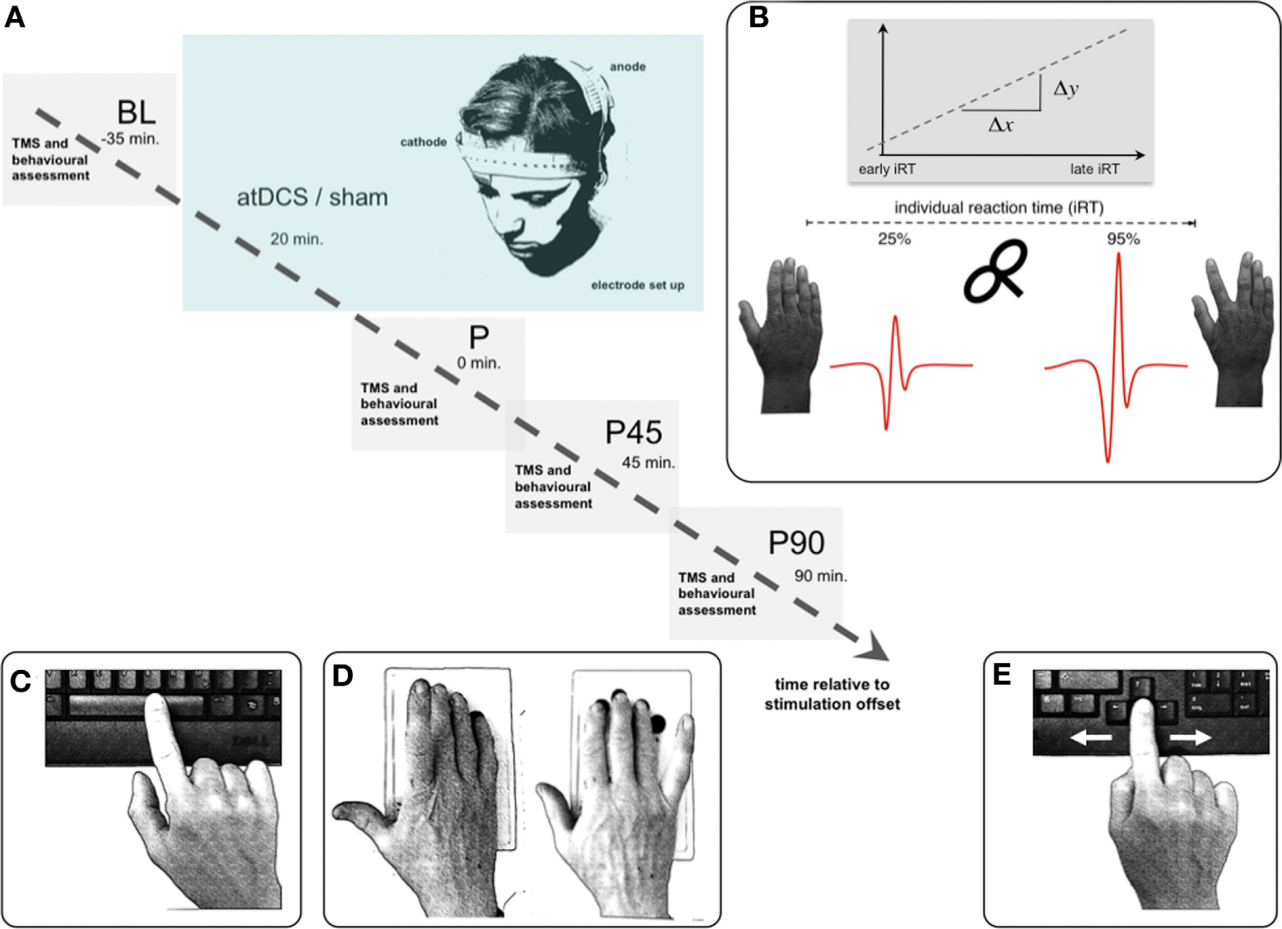
**Experimental setup. (A)** Volunteers participated in two separate sessions (anodal/sham stimulation), pseudorandomized within each age group. Time flow within each single (anodal/sham stimulation) session: resting-state and event-related TMS as well as behavioral measurements were performed immediately, 45, and 90 min (P, P45, P90) after 20 min of anodal or sham transcranial direct current stimulation. **(B)** TMS paradigm for *event-related* SICI_move_ measurement. Based on the individual reaction time (iRT), determined at the beginning of each measurement time point before the TMS experiment, unconditioned and conditioned TMS was applied early (~25% of iRT) and late (~90% of iRT) during the preparation of a visually paced index finger adduction for acquisition of *event-related* TMS data. A total of 16 pulses were applied at every measurement time point for each stimulation condition during *resting-state*, and per time zone during *event-related* measurement. **(C–E)** Behavioral measurements: Dexterous manual behavior was tested with three different task involving FDI activity with graded level of complexity tested in pseudorandomized order. **(C)** Solitary index finger tapping (1FT) and **(D)** alternating index and little finger tapping (2FT) were recorded over 3 × 10 s between GO- and STOP-signal. In both tasks the subjects were instructed to tap as fast and as precise as possible on pre-defined buttons of a 4-digit keypad. **(E)** During the choice-reaction time task (CRT) participants were asked to respond as fast as possible to a (neutrally pre-cued) target stimulus indicating either a left or a right index finger key press starting from a standardized middle position on a standard keyboard. Inter-trial intervals jittered between 1 and 6 s. For all experiments participants were seated with forearms supported on a table. Any whole hand, wrist, or arm movements were physically restricted throughout all behavioral experiments. Hand positioning on the keyboard assured movement of respective fingers only. Please note that the graphs depict the behavioral tasks without restriction for display purposes only.

The effect of atDCS on resting-state and event-related intracortical inhibition and behavior was tested in a double blind crossover design. Participants and all study personal involved in data acquisition and analysis were blind regarding the type of stimulation condition. All volunteers participated in two separate sessions (anodal/sham stimulation). The order of anodal vs. sham stimulation was pseudorandomized within each age group. A minimum interval of 48 h between sessions (range 2–33 days) assured complete wash-out of the single-session atDCS effect (Nitsche et al., 2008). Within each session, the measurement of electrophysiological and behavioral parameters was performed before, and at three time points—immediately, 45, and 90 min (P, P45, P90)—after stimulation (Figure 1A). The time points of measurement are based on previous findings showing physiological effects outlasting stimulation for up to 90 min (Nitsche and Paulus, 2001; Nitsche et al., 2003b). Attention and fatigue were repeatedly evaluated during each session at the beginning of every measurement time point (separate Visual Analog Scales).

### Transcranial direct current stimulation

Anodal trancranial direct current stimulation (atDCS) was delivered with an intensity of 1 mA for 20 min in the tDCS session using an eldith DC-Stimulator (neuroConn, Ilmenau, Germany) with two 25 cm^2^ saline-soaked gel-sponge electrodes (0.04 mA/cm^2^ current density). In accordance with established protocols (Nitsche et al., 2008), the anode was positioned over the representation of the first dorsal interosseus muscle (FDI) within the left MI, which was determined with single pulse TMS (Figure 1A). The cathode was placed on the skin overlying the contralateral supraorbital region (Nitsche and Paulus, 2000).

Anodal tDCS applied in this way results in an increase in excitability of the underlying MI that outlasts the period of stimulation (Lang et al., 2004). Sham stimulation was administered according to a well-established protocol (Gandiga et al., 2006). At the onset of both interventions (atDCS and Sham), current was increased in a ramp-like fashion eliciting a transient tingling sensation on the scalp that faded over seconds and that elicited comparable perceptions. Current remained at the 1 mA level for 20 min in the tDCS session and for up to 30 s in the Sham session. In both sessions, currents were turned off slowly over 8 s; a procedure that does not elicit perceptions and that was implemented out of the field of view of the subjects. During the application of stimulation participants were presented a video to enhance blinding and assure level of attention and alertness.

### Transcranial magnetic stimulation

Two Magstim 200 magnetic stimulators connected via one Bistim module (Magstim Company, Whitland, Dyfed, UK) and one figure-of-eight coil with 80 mm wing diameter were used for single and double pulse application. The coil was placed over the hand motor area, with the handle in antero-medial orientation, ~45° to the interhemispheric line. Procedures to establish motor hotspot for the first dorsal interosseus muscle (FDI) and resting motor threshold followed standardized procedures (Siebner and Rothwell, 2003). Resting motor threshold (rMT) was defined as the percentage of maximum of stimulator output (%MSO) to produce MEP amplitudes of at least 50 μV in five out of ten consecutive trials (Rossini et al., 1999). Subthreshold conditioning stimulus was followed by a suprathreshold test stimulus in the paired pulse paradigm. We used an interstimulus interval of 3 ms to evaluate SICI (Kujirai et al., 1993). Conditioning stimulus was set at 80% of resting motor threshold (Ziemann et al., 1996b) and the test stimulus was adjusted to elicit unconditioned MEP amplitudes of 0.5–1 mV peak-to-peak. Since the focus of the current work was set on evaluating the specific effect of atDCS on SICI, stimulus intensities (CS and TS) were re-adjusted prior to every measurement time point (BL, P, P45, P90).

Based on the individual reaction time (iRT), determined at the beginning of each measurement time point before the TMS experiment, unconditioned and conditioned TMS was applied early (~25% of iRT) and late (~90% of iRT) during the preparation of a visually paced index finger adduction for acquisition of *event-related* TMS data as described previously (Hummel et al., 2009; Heise et al., 2010, 2013) (Figure 1B). A total of 16 pulses were applied at every measurement time point for each stimulation condition during *resting-state*, and per time zone during event-related measurement.

EMG signals were recorded with disposable surface Ag/AgCl electrodes from the FDI in a belly-tendon montage, amplified, and digitized (CED MICRO 1401, Cambridge Electronic Design, Cambridge, UK) and electronically stored for off-line analysis.

### Behavioral data

Dexterous manual behavior was tested with three different tasks involving FDI activity with graded level of complexity as described previously (Heise et al., 2013).

Solitary index finger tapping (1FT, Figure 1C) and alternating index and little finger tapping (2FT, Figure 1D) were recorded over 3 × 10 s between GO- and STOP-signal. In both tasks the subjects were instructed to tap as fast and as precise as possible on pre-defined buttons of a 4-digit keypad. The order of behavioral tasks was pseudorandomized across session and participant within each measurement time point (BL, P, P45, P90).

During the choice-reaction time task (CRT, Figure 1E), participants were asked to respond as fast as possible to a (neutrally pre-cued) target stimulus indicating either a left or a right index finger key press starting from a standardized middle position on a standard keyboard. Inter-trial intervals jittered between 1 and 6 s.

Visual cues were provided on a 20-inch computer screen by Presentation software (Neurobehavioral Systems, Inc., Albany, CA, USA), also used to record response parameters (number of key presses, reaction time, key-press intervals, key selection) for off-line analyzes.

For all experiments participants were seated with forearms supported on a table. Any whole hand, wrist, or arm movements were physically restricted throughout all behavioral experiments. Hand positioning on the keyboard assured movement of respective fingers only. Participants were tested on the dominant right hand only.

### Control experiment

Since we were not able to exactly reproduce the atDCS effect on SICI of previous findings (Nitsche et al., 2005; Kidgell et al., 2013), who examined SICI with a CS adjusted to 70% of active motor threshold (aMT) and different stimulation intensities, we introduced a control experiment, consisting of two additional sessions. In young healthy subjects (*N* = 5, presenting not the expected release of inhibition after atDCS), SICI was measured with two different paradigms (setting 1: CS adjusted to 80%rMT, setting 2: CS adjusted to 70% aMT) before and after (immediately and 45 min) either 7 or 13 min of stimulation duration in two separate sessions (≥24 h inter-session interval, order counter-balanced). For each SICI setting and time point (BL, P, P45) 20 conditioned and 20 unconditioned stimuli were applied.

### Data processing

Any EMG-data contaminated with muscle activity before TMS pulses were discarded from further analysis after visual inspection. Motor-evoked potential (MEP) amplitudes were measured peak-to-peak. As it is standard practice SICI was normalized to the corresponding unconditioned MEP (SICI = conditioned MEP/unconditioned MEP × 100) at either resting-state (SICI_rest_) or respective pre-move time zones for event-related modulation (SICI_move_) (Hummel et al., 2009; Heise et al., 2010, 2013).

Behavioral data were processed using a customized automated log file parser to calculate outcome variables (1FT: response time for inter-tap interval, 2FT: response time for valid transitions between finger V and II, CRT: response time for correct key presses).

### Statistical analyzes

A random coefficient multilevel model was used for analysis of stimulation-induced change in *resting-state* (SICI_rest_) and *event-related* SICI modulation (SICI_move_), as well as in behavioral data (1FT, 2FT, CRT). Primary outcome was the stimulation-induced change (Δ) in all dependent variables (DV), normalized to baseline (ΔSICI_rest_, ΔSICI_move_, Δ1FT, Δ2FT, ΔCRT).

Stimulation induced change and its temporal pattern among age groups was the main focus of the analysis. Therefore, separate models were fitted for each DV with STIMULATION CONDITION (atDCS, sham), GROUP (old, young), and TIME POINT of measurement within each session (BL, P, P45, P90) as fixed factors.

Change in SICI induction during movement preparation (SICI_move_) was fitted as a linear trend from early to late pre-move phase and added as a covariate (pTIMEST) to the model, since this has been proven to adequately estimate *event-related* SICI modulation (Heise et al., 2013). Improved model fit was tested including random intercept for (i) SUBJECT and (ii) linear slope (pTIMEST) as random slope for the SICI_move_ model, since we expected a certain amount of intra-individual variance to be influenced by stimulation condition. In order to estimate variances of random effects, restricted maximum likelihood (REML) criteria were employed. Model selection was strictly hypothesis driven and dictated by the experimental design, therefore all fixed effects and interactions remained in the final model. Final model selection was based on Bayesian Information Criterion (BIC) for model comparison, normalized residuals based on REML fit served for model validation (Brown and Prescott, 2006; Pinheiro and Bates, 2009).

In order to exclude any crossover effect of stimulation on behavioral outcome, which we expected to change with constant practice although participants were familiarized to the tasks prior to the experiment, factor SEQUENCE (order of stimulation conditions over sessions) was introduced. Furthermore, to be able to differentiate the latter from a pure effect of training, SESSION (1st, 2nd) was also modeled as fixed effect as recommended for the analysis of longitudinal cross-over experiments (DíAz-Uriarte, 2002).

Since previous findings revealed an association between better event-related SICI modulation and more skilful manual performance (Heise et al., 2013), here principal component analysis (PCA) was used to extract the common information of “dexterous manual performance” (1FT, 2FT, CRT pooled for BL) and entered into correlation analysis with event-related SICI_move_ modulation (linear slope, pTIMEST) to verify this association. In a next step, the association between stimulation-induced change (*net* change, Δ_atDCS_ − Δ_sham_) in resting-state and event-related SICI, as well as respective association with net change in behavior was analyzed. Furthermore, the question was whether baseline event-related SICI modulation was associated with stimulation-induced change of resting-state SICI (SICI_move_ modulation at BL and ΔSICI_rest_ pooled for P and P45).

Since in the 2FT task, no stable level of performance was achieved but an obvious performance improvement (5–10% speed increase) was also observed in the sham condition, skill learning had to be assumed. Therefore, a secondary analysis of the learning curve in the 2FT task (outcome centered) was performed using a growth curve, i.e., modeling time (continuous) as linear trend, second and third order polynomial (poly[TIME 3]_linear, quadratic, cubic_), to estimate the curvilinear temporal pattern of performance change over time. As fixed factors STIMULATION CONDITION and AGE GROUP and their respective interactions with time were modeled. Random intercept and slope and STIMULATION CONDITION nested within subjects (~time | ID/STIMULATION CONDITION), were modeled using an autoregressive covariance structure: AR(1). Effect sizes of atDCS on individual learning curves, using random effects of the growth curve model (representing the individual deviation from the estimated subgroup mean), were correlated (partial correlation correcting for age group) with atDCS-induced (net) change of SICI_rest_ and SICI_move_ modulation.

### Control experiment

Stimulation-induced change in SICI (ΔSICI: conditioned/unconditioned MEP amplitude^*^100, expressed as difference from BL) was analyzed with fixed factors TIME POINT (P, P45), STIMULATION DURATION (7, 13 min), and SICI SETTING (CS 70%aMT, CS 80%rMT) and tested for their interaction. Modeling random intercept for SUBJECT improved model fit.

Data cleaning was kept to a minimum, as recommended for reaction time data (Baayen and Milin, 2010), only excluding physically impossible trials (e.g., CRT <100 ms). Each part of the analysis was carried out on the maximum available data set. Missing data were not replaced or imputed, neither in outcome nor in independent variable or covariates. Data preparation and statistical analyzes were performed using the software package R for Statistical Computing version 2.15.1 (2012-06-022, www.r-project.org/) for Mac OS X GUI 1.40-devel Leopard build 64-bit, package nlme version 3.1-104 for linear mixed effects modeling (Pinheiro et al., 2012), package multcomp version 1.2-12 for simultaneous *post-hoc* pairwise comparison and respective corrections (Hothorn et al., 2008). Partial correlations (Kendall's τ) adjusting for factor for AGE GROUP were calculated using ppcor package version 1.0 (Kim, 2012), multiple correlations were computed using percentage-bend correlations (Wilcox, 2012), adjusted correlation coefficients (r_pb_), percentile bootstrap 95% confidence intervals for correlation coefficients, value of test statistics (T_pb_), and level of significance are given.

All graphical presentation of data is done with packages lattice version 0.20-6 (Sarkar, 2008) and ggplot2 version 0.9.1 (Wickham, 2009). Results for random coefficient models are given as Type III sums of squares for sequentially fitted fixed effects (F, df, p), Wald statistics for marginal parameter estimates (t, df, p, 95% CI), as well as variance component estimates for random effects (variance, SD). Significant results from simultaneous pairwise *post-hoc* comparison with Tukey Contrast are given with adjusted *p*-value for estimates of contrasts (estimate ± SE, *z*-value, adjusted p).

## Results

### Change in resting-state SICI (ΔSICI_rest_)

Average SICI_rest_ and ΔSICI_rest_ are given in Table 1 for stimulation conditions nested within age group per time point. Absolute resting-state SICI was significantly diminished (less inhibition) in older subjects (for details in analysis of absolute SICI refer to supplemental online results).

**Table 1:** **Descriptive data for resting-state and event-related SICI for stimulation conditions within age group for separate time point**.

**Time point**	**Age group**	**Stimulation condition**	**SICI_rest_ (in %)**	**ΔSICI_rest_**	**SICI_move_ slope (in %)**	**change in SICI_move_ slope (ΔpTIMEST)**
BL	OLDER	atDCS	86.17 ± 5.82		16.97 ± 6.35	
		Sham	90.81 ± 8.86		15.35 ± 4.97	
	YOUNGER	atDCS	50.80 ± 4.27		58.97 ± 7.78	
		Sham	34.06 ± 2.50		62.08 ± 8.01	
P	OLDER	atDCS	64.75 ± 4.15	−26.24 ± 3.23	10.86 ± 8.65	163.38 ± 153.50
		Sham	98.23 ± 8.51	3.96 ± 7.81	10.29 ± 9.38	−199.17 ± 259.56
	YOUNGER	atDCS	40.87 ± 3.04	3.83 ± 3.44	19.31 ± 3.03	−24.86 ± 62.20
		Sham	41.88 ± 3.53	11.29 ± 0.66	19.77 ± 3.06	35.03 ± 6.60
P45	OLDER	atDCS	69.00 ± 4.43	−21.30 ± 5.30	2.15 ± 6.73	105.54 ± 93.43
		Sham	75.60 ± 6.90	−16.93 ± 4.59	6.14 ± 5.98	−102.10 ± 199.89
	YOUNGER	atDCS	49.75 ± 3.18	13.13 ± 2.84	20.26 ± 2.91	9.85 ± 21.52
		Sham	41.98 ± 2.66	12.17 ± 2.41	21.41 ± 3.21	42.37 ± 8.33
P90	OLDER	atDCS	69.40 ± 5.16	−22.81 ± 4.68	12.43 ± 2.82	63.69 ± 34.96
		Sham	90.18 ± 9.08	−4.71 ± 10.13	16.18 ± 2.65	−23.65 ± 144.04
	YOUNGER	atDCS	68.10 ± 10.47	31.14 ± 10.56	18.12 ± 1.51	−56.49 ± 64.89
		Sham	43.75 ± 2.70	13.01 ± 2.51	18.33 ± 1.20	41.06 ± 7.06

**Figure 2:**
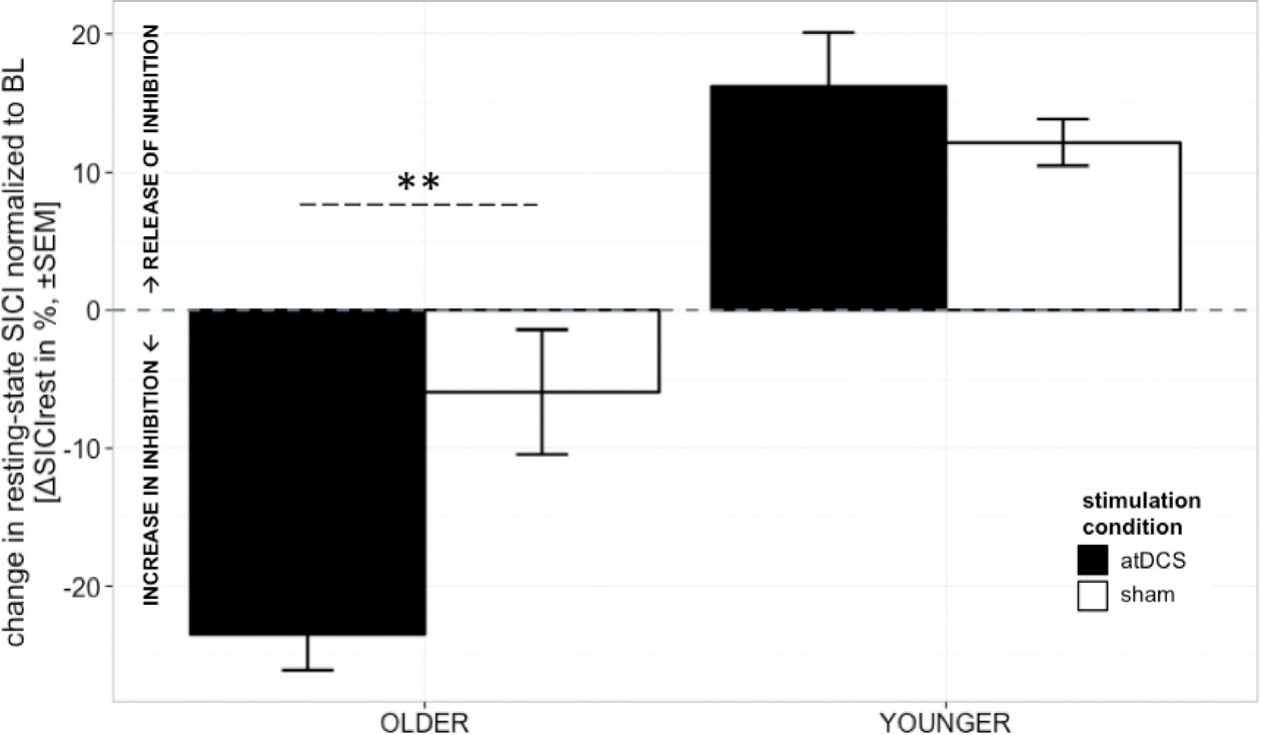
Change in resting-state SICI (ΔSICI_rest_, conditioned MEP/unconditioned MEP amplitude^*^100 normalized to BL) was distinguishable among age groups. Older participants showed an increase of inhibition under atDCS compared to the sham condition (*p* < 0.005), while younger participants tended to show disinhibition after both stimulation conditions. The differential atDCS effect tended to be modulated by time, immediately present after stimulation cessation in the older group but rising in extent with increasing time after stimulation in the younger group (*p* = 0.06). ^***^p < 0.001, ^**^p < 0.01, ^*^p < 0.05, °*p* < 0.1.

A clear differentiation of the effect of atDCS on change in resting-state SICI (ΔSICI_rest_) was found between age groups [STIMULATION CONDITION × AGE GROUP, *F*_(1, 2879)_ = 10.80, *p* < 0.001, Figure 2, Tables 2 and 3]. While the old group showed overall a significant increase of inhibition (more inhibition) under atDCS compared to the sham condition (older subgroup, sham vs. atDCS: 13.51 ± 3.82, *z* = 3.54, *p* < 0.005), there was no significant contrast between stimulation conditions in the young group, showing a trend of disinhibition after both stimulation conditions (younger subgroup, sham vs. atDCS: −4.22 ± 3.75, *z* = −1.13, *p* > 0.6). Furthermore, there was a trend for time to modulate this interaction [TIME POINT × STIMULATION CONDITION × AGE GROUP, *F*_(2, 2879)_ = 2.85, *p* = 0.058], parameter estimates indicated that the differential atDCS effect was immediately present after stimulation cessation in the older group but increased after stimulation in the younger group. The necessity of adjusting for significant intra-individual variability in ΔSICI_rest_ was shown by improved model fit (smaller BIC) with modeling a random intercept for subject.

**Table 2:** **Models for change in SICI resting-state and even-related SICI (DV: ΔSICI_rest_, ΔSICI_move_)**.

**Coefficients**	**ΔSICI_rest_**		**ΔSICI_move_**	
	**Estimate (*SE*)**	***t*-value (*df*)**	**Estimate (*SE*)**	***t*-value (*df*)**
Intercept	11.17 (16.12)	0.69 (2879)	35.03 (119.13)	0.29 (150)
TIME POINT (P45)	0.75 (6.50)	0.12 (2879)	7.34 (165.65)	0.04 (150)
TIME POINT (P90)	1.77 (6.43)	0.28 (2879)	6.03 (165.65)	0.036 (150)
STIMULATION CONDITION (atDCS)	−7.40 (6.50)	−1.14 (2879)	−59.90 (165.65)	−0.36 (150)
AGE GROUP (older)	−8.23 (22.81)	−0.36 (30)	−234.20 (165.65)	−1.39 (30)
TIME POINT (P45) STIMULATION CONDITION (atDCS)	8.57 (9.19)	0.93 (2879)	27.37 (234.27)	0.12 (150)
TIME POINT (P90) × STIMULATION CONDITION (atDCS)	25.66 (9.14)	2.81^**^ (2879)	−37.65 (234.27)	−0.16 (150)
TIME POINT (P45) × AGE GROUP (older)	−16.18 (9.22)	−1.76° (2879)	89.73 (234.27)	0.38 (150)
TIME POINT (P90) × AGE GROUP (older)	−5.91 (9.18)	−0.64 (2879)	169.49 (234.27)	0.72 (150)
STIMULATION CONDITION (atDCS) × AGE GROUP (older)	−19.24 (9.22)	−2.09^*^ (2879)	422.44 (234.27)	1.80° (150)
TIME POINT (P45) × STIMULATION CONDITION (atDCS) × AGE GROUP (older)	18.26 (13.09)	1.40 (2879)	−182.28 (331.30)	−0.55 (150)
TIME POINT (P90) × STIMULATION CONDITION (atDCS) × AGE GROUP (older)	−12.81 (13.02)	−0.98 (2879)	−237.55 (331.30)	−0.72 (150)
**Random effect: ~1|ID**	**Variance (*SD*)**			
(Intercept)	3826.97 (61.86)		7533.91 (86.80)	
Residual	5186.86 (72.02)		219,520.16 (468.53)	

^***^p < 0.001,

** p < 0.01,

* p < 0.05,

° p< 0.1.

**Table 3:** ***F*-tests (type III sums of squares) for change of resting-state and even-related SICI (DV: ΔSICI_rest_, ΔSICI_move_)**.

**Coefficients**	***F*-value (*df*)**	
	**ΔSICI_rest_**	**ΔSICI_move_**
Intercept	0.03 (1, 2879)	0.02 (1, 150)
TIME POINT	3.93^*^ (2, 2879)	0.03 (2, 150)
STIMULATION CONDITION	2.86 (1, 2879)	1.33 (1, 150)
AGE GROUP	1.22 (1, 30)	0.01 (1, 30)
TIME POINT × STIMULATION CONDITION	5.44^**^ (2, 2879)	0.45 (2, 150)
TIME POINT × AGE GROUP	1.82 (2, 2879)	0.06 (2, 150)
STIMULATION CONDITION × AGE GROUP	10.80^***^ (1, 2879)	4.36^*^ (1, 150)
TIME POINT × STIMULATION CONDITION × AGE GROUP	2.85° (2, 2879)	0.28 (2, 150)

*** p < 0.001,

** p < 0.01,

* p < 0.05,

° p < 0.1.

### Change in event-related SICI (ΔSICI_move_)

**Figure 3:**
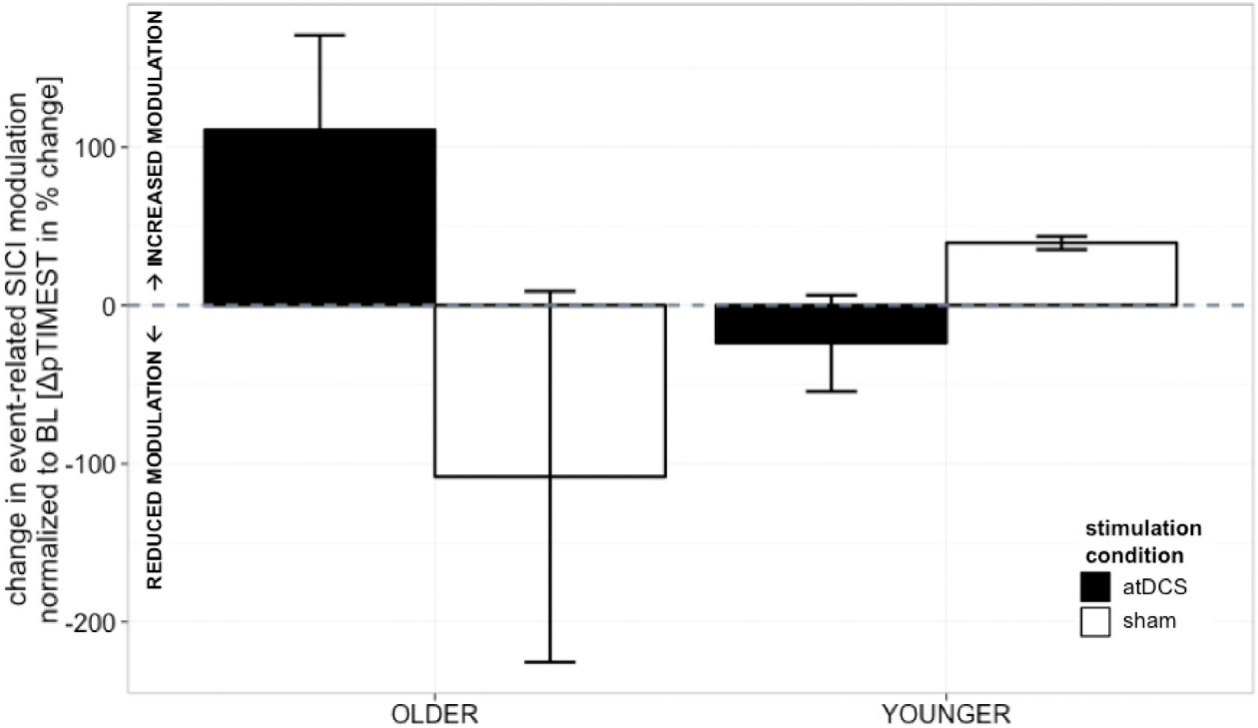
**Change in event-related SICI modulation (linear slope normalized to BL slope, in%, ±s.e.m.)**. Older participants showed a non-significant trend toward increase in modulatory capacity after atDCS (*p* = 0.09) while modulation remained unaffected by stimulation condition in the younger group (*p* > 0.9). Black indicates change in atDCS condition, white depicts sham condition. ^***^p < 0.001, ^**^p < 0.01, ^*^p < 0.05, °*p* < 0.1.

Absolute event-related SICI modulation was drastically diminished in the old group irrespective of stimulation condition or time point of measurement as demonstrated previously (Heise et al., 2013) (Table 1, details are given in supplemental online results). A significant effect of stimulation on change in SICI_move_ slope was found to diverge among age groups [STIMULATION CONDITION × AGE GROUP, *F*_(1, 150)_ = 4.36, *p* < 0.05, ΔSICI_move_, Tables 2 and 3]. In the older group, a trend toward increase in modulatory capacity after atDCS was found (OLDER subgroup, sham vs. atDCS: −219.2 ± 95.6, *z* = −2.29, *p* = 0.09) while modulation remained unaffected by stimulation condition in the younger group (YOUNGER subgroup, sham vs. atDCS: 63.32 ± 95.6, *z* = 0.66, *p* > 0.9, Figure 3).

### Behavioral data

Detailed results for absolute behavioral performance are given in supplemental online material. Here, only results for main outcome, i.e., change in behavior normalized to BL (Δ1FT, Δ2FT, ΔCRT), are given.

### Change in 1FT (Δ1FT)

Performance in 1FT task was marked by a significant performance decrease, i.e., increase of intertap response time, over time (Table 4).

**Table 4:** **Descriptive data for behavioral measures 1FT, 2FT, and CRT (absolute in ms and change Δin%) given as mean ± s.e.m. for stimulation conditions nested within age group per time point**.

**Time point**	**Age group**	**Stimulation condition**	**1FT**		**2FT**		**CRT**	
			**1FT (in ms)**	**Δ1FT (in %)**	**2FT (in ms)**	**Δ2FT (in %)**	**CRT (in ms)**	**ΔCRT (in %)**
BL	OLDER	atDCS	201.08 ± 0.76		299.64 ± 5.10		655.10 ± 7	
		Sham	200.53 ± 1.22		283.53 ± 4.58		640.34 ± 8.02	
	YOUNGER	atDCS	180.71 ± 0.66		127.17 ± 1.40		453.81 ± 3.28	
		Sham	181.47 ± 0.70		123.98 ± 1.53		451.79 ± 2.88	
P	OLDER	atDCS	201.49 ± 0.76	−0.44 ± 0.76	264.35 ± 3.92	−11.10 ± 0.89	629.69 ± 5.89	−3.03 ± 0.72
		sham	206.24 ± 0.98	2.85 ± 0.98	261.97 ± 4.00	−5.78 ± 0.91	636.75 ± 7.74	−0.93 ± 1.00
	YOUNGER	atDCS	184.13 ± 0.80	2.59 ± 0.80	114.50 ± 1.49	−7.19 ± 1.06	438.87 ± 3.09	−3.10 ± 0.60
		sham	183.85 ± 0.76	1.31 ± 0.76	111.60 ± 1.55	−8.76 ± 1.09	455.89 ± 4.15	−2.92 ± 1.15
P45	OLDER	atDCS	202.25 ± 0.81	−0.06 ± 0.81	256.90 ± 4.49	−15.88 ± 0.86	637.39 ± 6.36	−2.04 ± 0.85
		sham	207.26 ± 0.91	3.37 ± 0.91	255.90 ± 4.43	−8.72 ± 1.00	630.58 ± 7.17	−0.92 ± 0.96
	YOUNGER	atDCS	187.34 ± 0.94	4.21 ± 0.94	111.05 ± 1.21	−8.95 ± 0.99	447.10 ± 3.40	−1.34 ± 0.67
		sham	186.36 ± 0.94	2.69 ± 0.94	107.35 ± 1.30	−11.51 ± 0.92	445.28 ± 3.03	−4.91 ± 1.01
P90	OLDER	atDCS			252.96 ± 4.45	−15.91 ± 1.06	634.28 ± 7.07	−2.13 ± 1.18
		sham			250.81 ± 3.98	−11.62 ± 1.09	644.14 ± 7.11	1.11 ± 0.96
	YOUNGER	atDCS			100.24 ± 1.19	−16.76 ± 0.95	439.94 ± 3.12	−2.95 ± 0.59
		sham			109.47 ± 1.45	−10.76 ± 0.98	438.33 ± 3.23	−6.61 ± 0.97

Note that for 1FT no data was acquired at time point P90.

**Figure 4:**
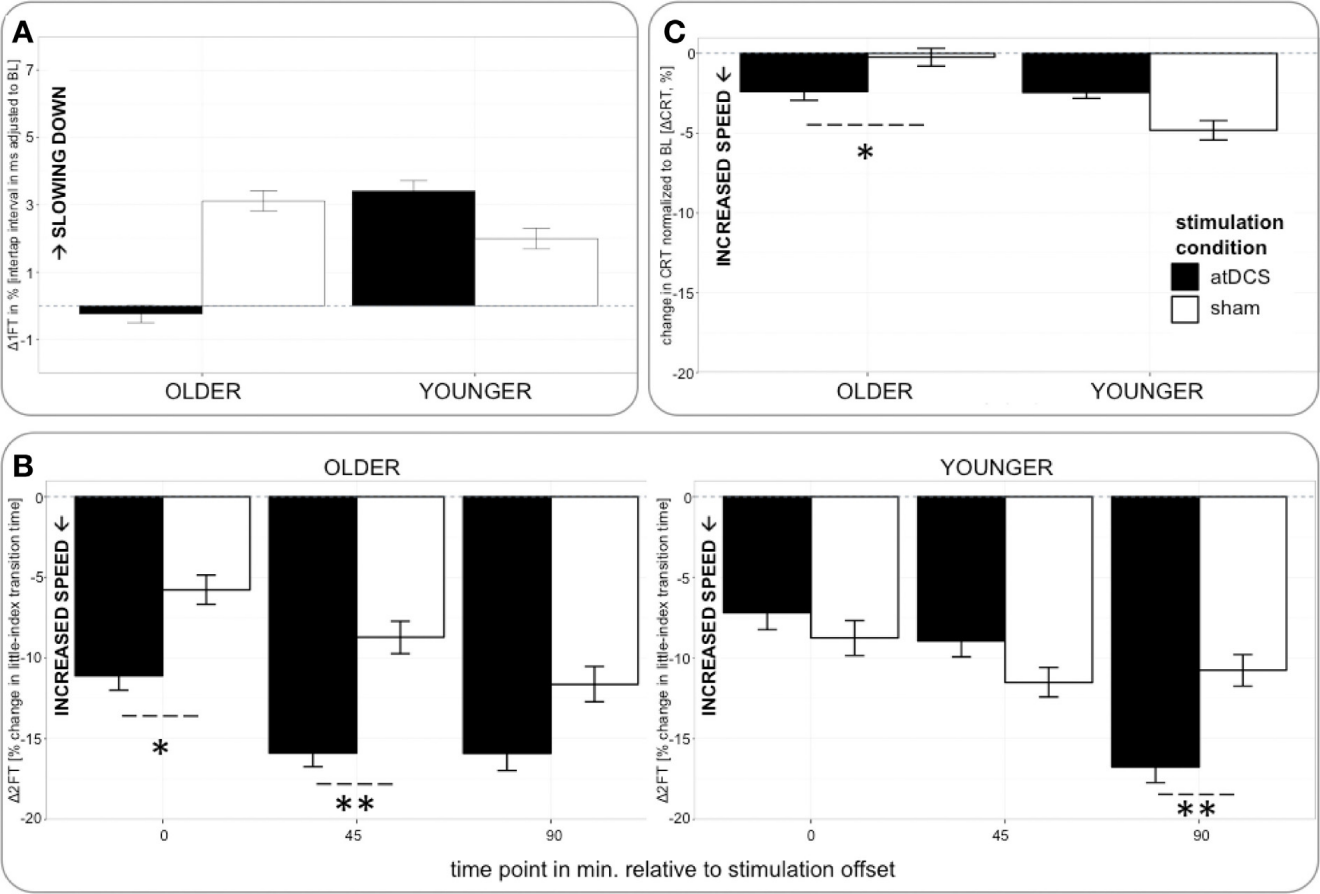
**(A)** (upper row, left) Change in 1FT normalized to BL (Δ1FT) over time. 1FT was marked by performance decrements in younger participants under any condition and older participants under sham condition. Performance tended to remain stable in older under atDCS. **(B)** (bottom) Change in 2FT normalized to BL (Δ2FT) over time. Older participants showed clear advantage under atDCS early after stimulation, at P and P45, while younger participants benefitted from atDCS at late time point P90. **(C)** (upper row right) Change in CRT normalized to BL (ΔCRT) over time. Older participants showed stable performance under sham and overall increasing response speed under atDCS. Left column depicts old subgroup, right column shows young subgroup, black indicates change in atDCS condition, and white depicts sham condition. ^***^p < 0.001, ^**^p < 0.01, ^*^p < 0.05, °*p* < 0.1.

Analyzing change in 1FT (Δ1FT, Tables 5 and 6) showed an increasing change in intertapping time, i.e., worsening of 1FT performance over time, revealed by a main effect of time point [*F*_(1, 19197)_ = 12.76, *p* < 0.001]. Furthermore, a main effect of stimulation condition [*F*_(1, 19197)_ = 6.14, *p* < 0.05], which was modulated by age group [AGE GROUP × STIMULATION CONDITION, *F*_(1, 19197)_ = 69.73, *p* < 0.0001]. Parameter estimates indicated that performance decrements were evident in younger participants under any condition and older participants under sham condition but performance tended to remain stable in older under atDCS, however, not confirmed by *post-hoc* pairwise testing (OLDER subgroup, atDCS vs. sham: 3.14 ± 1.51, *z* = 2.08, *p* > 0.1, atDCS subgroup, OLDER vs. YOUNGER: 4.17 ± 2.22, *z* = 1.88, *p* > 0.2; all other contrasts *p* > 0.7, Figure 4A).

**Table 5:** **Wald statistics for performance change in behavioral measures (DV: Δ1FT, Δ2FT, ΔCRT) model**.

**Coefficients**	**Δ1FT^§^**		**Δ2FT**		**ΔCRT**	
	**Estimate (*SE*)**	***t*-value (*df*)**	**Estimate (*SE*)**	***t*-value (*df*)**	**Estimate (*SE*)**	***t*-value (*df*)**
(Intercept)	1.60 (1.41)	1.14 (19197)	−9.57 (2.77)	−3.46^***^ (14078)	−2.48 (2.02)	−1.23 (6276)
AGE GROUP (older)	1.10 (2.00)	0.55 (30)	5.58 (4.00)	1.39 (30)	2.0 (2.87)	0.70 (30)
TIME POINT (P45)	1.41 (0.56)	2.51^*^ (19197)	−3.04 (1.22)	−2.49^*^ (14078)	−1.98 (1.19)	−1.66° (6276)
TIME POINT (P90)			−1.77 (1.23)	−1.45 (14078)	−3.670 (1.19)	−3.10^**^ (6276)
STIMULATION CONDITION (atDCS)	1.44 (0.56)	2.56^*^ (19197)	1.29 (1.24)	1.04 (14078)	−1.39 (1.20)	−1.16 (6276)
TIME POINT (P45) × AGE GROUP (older)	−0.83 (0.82)	−1.02 (19197)	0.40 (2.08)	0.19 (14078)	2.12 (1.72)	1.24 (6276)
TIME POINT (P90) × AGE GROUP (older)			−4.07 (2.07)	−1.97^*^ (14078)	5.88 (1.71)	3.43^***^ (6276)
STIMULATION CONDITION (atDCS) × AGE GROUP (older)	−4.57 (0.82)	−5.60^***^ (19197)	−7.28 (2.11)	−3.45^***^ (14078)	−0.57 (1.72)	−0.33 (6276)
TIME POINT (P45) × STIMULATION CONDITION (atDCS)	0.28 (0.80)	0.35 (19197)	1.00 (1.75)	0.58 (14078)	3.79 (1.69)	2.25^*^ (6276)
TIME POINT (P90) × STIMULATION CONDITION (atDCS)			−7.89 (1.74)	−4.52^***^ (14078)	3.90 (1.69)	2.31^*^ (6276)
TIME POINT (P45) × STIMULATION CONDITION (atDCS) × AGE GROUP (older)	−0.50 (1.15)	−0.43 (19197)	−2.41 (2.96)	−0.82 (14078)	−2.91 (2.42)	−1.20 (6276)
TIME POINT (P90) × STIMULATION CONDITION (atDCS) × AGE GROUP (older)			9.77 (2.95)	3.31^***^ (14078)	−5.20 (2.42)	−2.15^*^ (6276)
**Random effect: ~1|ID**	**Variance (*SD*)**					
(Intercept)	29.19 (5.40)		110.5375 (10.51)		53.71 (7.33)	
Residual	398.39 (19.96)		1158.1526 (34.03)		383.55 (19.58)	

§ 1FT data was not acquired at time point P90,

*** p < 0.001,

** p < 0.01,

* p < 0.05,

° p < 0.1.

**Table 6:** ***F*-tests (type III sums of squares) for performance change in behavioral measures (DV: Δ1FT, Δ2FT, ΔCRT)**.

**Coefficients**	***F*-value (*df*)**		
	**Δ1FT**	**Δ2FT**	**ΔCRT**
(Intercept)	5.26^*^ (1, 19197)	32.46^***^ (1, 14078)	2.96 (1, 6276)
AGE GROUP	0.80 (1, 30)	0.28 (1, 30)	1.31 (1, 30)
TIME POINT	12.76^***^ (1, 19197)	29.80^***^ (2, 14078)	0.20 (2, 6276)
STIMULATION CONDITION	6.14^*^ (1, 19197)	22.33^***^ (1, 14078)	0.77 (1, 6276)
TIME POINT × AGE GROUP	3.62° (1, 19197)	0.60 (2, 14078)	4.18 (2, 6276)
STIMULATION CONDITION × AGE GROUP	69.73^***^ (1, 19197)	15.78^***^ (1, 14078)	10.93*** (1, 6276)
TIME POINT × STIMULATION CONDITION	0.004 (1, 19197)	7.06^***^ (2, 14078)	1.95 (2, 6276)
TIME POINT × STIMULATION CONDITION × AGE GROUP	0.19 (1, 19197)	9.68^***^ (2, 14078)	2.32° (2, 6276)

*** p < 0.001,

^**^p < 0.01,

* p < 0.05,

° p < 0.1.

### Change in 2FT (Δ2FT)

Average transition times of absolute 2FT are given in Table 4. While young participants were faster, there was an overall increase in performance speed.

The analysis of change in 2FT performance (Δ2FT, Tables 5 and 6) revealed a differentiation of performance change between age groups over time, which was significantly modulated by stimulation condition [STIMULATION CONDITION × TIME POINT × AGE GROUP, *F*_(2, 14078)_ = 9.68, *p* < 0.0001, Table 5]. Simultaneous pairwise *post-hoc* testing confirmed a significant difference in performance change between stimulation conditions showing a clear advantage under atDCS in older participants early after stimulation, at time point P (sham vs. atDCS 5.99 ± 1.70, *z* = 3.52, *p* < 0.05) and P45 (sham vs. atDCS 7.40 ± 1.67, *z* = 4.42, *p* < 0.01), but not later at time point P90 (sham vs. atDCS 4.11 ± 1.67, *z* = 2.47, *p* > 0.2). In younger participants, no difference in performance change between stimulation conditions was found early after stimulation cessation, at P (sham vs. atDCS −1.29 ± 1.24, *z* = −1.04, *p* > 0.9) or P45 (sham vs. atDCS −2.30 ± 1.23, *z* = −1.87, *p* > 0.7), but at late time point P90 performance increase was larger under atDCS (sham vs. atDCS 6.60 ± 1.22, *z* = 5.39, *p* < 0.01, Figure 4B).

### Change in CRT (ΔCRT)

Overall, participants performed with an average response time of 544.6 ms in the CRT, corresponding to an intercept of 6.3 ± 0.02 on the log scale. Younger participants were on average 6% faster and a significant decrease of average CRT performance time was observable over time (Table 4).

Analyzing change in CRT (ΔCRT, Tables 5 and 6) revealed a differentiation in the stimulation effect among age group [AGE GROUP × STIMULATION CONDITION, *F*_(1, 6276)_ = 10.93, *p* < 0.001, Table 5]. Simultaneous pairwise *post-hoc* testing verified that this effect was driven by the older group, who showed stable performance under sham and overall increasing response speed under atDCS (sham vs. atDCS 2.11 ± 0.71, *z* = 2.98, *p* < 0.05, Figure 4C). Furthermore, performance change over time differed among age groups [AGE GROUP × TIME POINT, *F*_(2, 6276)_ = 4.18, *p* < 0.05], with decreasing performance improvement in older and increasing performance in younger. However, this effect was small and did not survive *post-hoc* testing (all pairwise contrasts *p* > 0.2).

### Correlation between initial SICI_move_ modulation and “dexterous manual performance”

Correlation analysis between “dexterous manual performance” (expressed as main component from PCA) and SICI_move_ revealed an association between more pronounced SICI_move_ modulation and more dexterous performance at baseline [τ = −0.56, *T*_(30)_ = −6.63, *p* < 0.01].

### Correlation between atDCS-induced changes in resting-state SICI and behavior (pooled over P and P45)

Net change in SICI_rest_ was not associated with net change in 1FT [partial correlation, τ = −0.14, *p* > 0.2, *T*_(29)_ = −1.11]. However, in the younger group, more pronounced net disinhibition was associated with net increase (faster) in 1FT performance [*r*_pb_ = −0.58, *CI* = −0.85 − −0.13, *T*_pb(14)_ = −2.67, *p* < 0.05], while no association was observed in the older group [*r_pb_* = −0.03, *CI* = −0.46 − 0.61, *T_pb_*_(14)_ = −2.67, *p* > 0.8]. In the case of 2FT, overall a weak trend was seen for net increase in inhibition to be associated with net decrease in performance, i.e., relative increase of performance time [partial correlation, τ = −0.19, *p* > 0.1, *T*_(29)_ = −1.4]. This direction in the association was mainly driven by the older age group, however not significant [older: *r*_pb_ = −0.42, *CI* = −0.77 − 0.11, *T*_pb(14)_ = −1.74, *p* = 0.1; younger: *r_pb_* = 0.08, *CI* = −0.57 − 0.62, *T*_pb(14)_ = 0.30, *p* > 0.7]. Stimulation-induced change in CRT performance showed overall a moderate association with change in resting-state SICI [partial correlation, τ = 0.29, *p* < 0.05, *T*_(29)_ = 2.32], indicating net inhibition to correlate with net performance increase, i.e., faster CRT. This association tended to be more pronounced in the older [older: *r_pb_* = 0.48, *CI* = −0.15 − 0.80, *T*_pb(14)_ = 2.02, *p* = 0.09; younger: *r_pb_* = 0.44, *CI* = −0.09 − 0.75, *T*_pb(14)_ = 1.84, *p* = 0.09], however not reaching level of significance when analyzed for age groups separately.

### Correlation between stimulation-induced change in event-related SICI modulation and change in behavior

No association between net change in event-related SICI modulation (ΔpTIMEST_atDCS_ − ΔpTIMEST_sham_) and net change in behavior was found.

### Correlation stimulation-induced change in resting-state and event-related SICI modulation

No association was found between stimulation-induced net change in resting-state and event-related SICI, neither when pooled over all time points, nor when analyzed for single time point.

### Correlation between initial SICI_move_ modulation and atDCS-induced ΔSICI_rest_

However, initial event-related SICI modulation might serve as potential predictor for stimulation-induced change in resting-state SICI. Partial correlation (controlling for AGE GROUP) between event-related SICI modulation at BL and atDCS-induced ΔSICI_rest_ pooled over all time points after stimulation revealed a marginally significant association between event-related SICI modulation and stimulation induced change in resting-state SICI [τ = 0.24, *T*_(29)_ = 1.86, *p* = 0.06], indicating more pronounced atDCS-induced SICI effect with better modulatory capacity of event-related SICI. Analyzing age groups separately revealed, that this association was mainly driven by the older group [*r_pb_* = 0.53, *CI* = −0.04 − 0.87, *T_pb_*_(14)_ = 2.36, *p* = 0.06], while younger did not show an association [*r_pb_* = 0.10, *CI* = −0.43 − 0.69, *T_pb_*_(14)_ = 0.39, *p* > 0.6, Figure S1A].

### Secondary analysis of stimulation effects on learning curve in 2FT

Growth curve analysis was used to estimate the influence of STIMULATION CONDITION and AGE GROUP on performance improvement in the 2FT task over time, i.e., learning curve. The overall temporal pattern of 2FT learning curve was curvilinear and best fitted by a second and a third order polynomial [poly[TIME 3], *F*_(1, 18557)_ = 429.12, *p* < 0.0001]. Learning curve varied between the two age groups [AGE GROUP × poly[TIME 3], *F*_(3, 18557)_ = 62.08, *p* < 0.0001], and was differentially influenced by stimulation condition [STIMULATION CONDITION × poly[TIME 3], *F*_(3, 18557)_ = 6.02, *p* < 0.0004]. Most important, the temporal pattern of the learning curve was differentially modulated by stimulation condition among the age groups [AGE GROUP × STIMULATION CONDITION × poly[TIME 3], *F*_(2, 18557)_ = 6.09, *p* < 0.0004, Table 7, for Wald statistics on parameter estimates refer to supplemental material]. Parameter estimates indicate that no difference regarding the linear trend was found, i.e., no difference in the linear slope among age groups irrespective of stimulation [triple interaction with linear trend: *t*_(18557)_ = −0.08, *p* > 0.9]. However, the temporal pattern of the learning curve varied in terms of concavity [triple interaction with quadratic trend: *t*_(18557)_ = 3.19, *p* < 0.01], and change of curvature [triple interaction with cubic trend: *t*_(18557)_ = 2.76, *p* < 0.01] showing that older participants showed larger improvements under atDCS early on while younger participants showed increase in learning curve steepness later on (Figure S2A).

**Table 7:** ***F*-test (type III sums of squares) learning curve 2FT**.

**Coefficients**	***F*-value (*df*)**
(Intercept)	51.55^***^ (1, 18, 557)
AGE GROUP	4.09° (1, 30)
STIMULATION CONDITION	0.15 (1, 30)
poly[TIME 3]	429.12^***^ (3, 18, 557)
STIMULATION CONDITION × AGE GROUP	0.001 (1, 30)
AGE GROUP × poly[TIME 3]	62.08*** (3, 18, 557)
STIMULATION CONDITION × poly[TIME 3]	6.0148*** (3, 18, 557)
STIMULATION CONDITION × AGE GROUP × poly[TIME 3]	6.0933^***^ (3, 18, 557)

*** p < 0.001,

^**^p < 0.01,

^*^p < 0.05,

° p < 0.1.

Correlation with net change in SICI_move_ modulation indicated that steeper learning curves in terms of more pronounced improvement, i.e., reduction of 2FT performance time, to be moderately associated with net reduction of SICI_move_ modulation [τ = 0.32, *T*_(29)_ = 2.51, *p* < 0.05]. This association was mainly driven by the younger subgroup [younger: *r_pb_* = 0.61, *CI* = −0.04 − 0.91, *p* < 0.05, older: *r_pb_* = 0.08, *CI* = −0.53 − 0.76, *p* > 0.6]. No association was found for net change of SICI_rest_ and learn curve effect size [τ = 0.21, *T*_(29)_ = 1.63, *p* = 0.1].

### Control experiment

Using a weaker CS of 70%aMT induced less inhibitory effect than a CS of 80%rMT (Table 8).

**Table 8:** Average SICI in control experiments (conditioned MEP/unconditioned MEP ^*^100, mean ± s.e.m.).

**Stimulation duration**	**Time point**	**Setting (1: 80%rMT, 2: 70%aMT)**	**SICI (in %)**
7 min	BL	1	35.69 ± 3.55
		2	74.30 ± 4.86
	P	1	29.93 ± 2.89
		2	75.36 ± 5.09
	P45	1	28.94 ± 2.76
		2	71.64 ± 4.31
13 min	BL	1	37.39 ± 3.13
		2	59.79 ± 4.74
	P	1	32.61 ± 2.70
		2	72.57 ± 6.28
	P45	1	43.66 ± 4.03
		2	66.75 ± 4.33

Analyzing the effect of atDCS on ΔSICI measured with different paradigms (setting 1: CS adjusted to 80%rMT, setting 2: CS adjusted to 70%aMT) and stimulation durations (7, 13 min.) over time (BL, P, P45) revealed a significant main effect of SETTING (*p* < 0.01) with setting 2 inducing on average 6.65% release of inhibition. Overall, STIMULATION DURATION was a significant main effect (*p* < 0.0005), with longer stimulation duration inducing more release of inhibition in contrast to shorter stimulation duration (Tables 9, 10).

**Table 9:** **Wald statistics control experiment (DV: ΔSICI)**.

**Coefficients**	**Estimate (Std. Error)**	***t*-value (df)**
Intercept	−5.70 (4.12)	−1.38 (764)
TIME POINT (P45)	−1.06 (4.81)	−0.22 (764)
STIMULATION DURATION (13 min)	1.04 (4.78)	0.22 (764)
SETTING (2)	6.65 (4.77)	1.40 (764)
TIME POINT (P45) × STIMULATION DURATION (13 min)	11.80 (6.75)	1.75 (764)
TIME POINT (P45) SETTING (2)	−3.25 (6.76)	−0.48 (764)
STIMULATION DURATION (13 min) × SETTING (2)	9.65 (6.73)	1.43 (764)
TIME POINT (P45) × STIMULATION DURATION (13 min) × SETTING (2)	−12.82 (9.54)	−1.35 (764)
**Random effects: ~time|ID**	**Variance (*SD*)**	
(Intercept)	0.02 (0.15)	
Residual	0.51 (0.72)	

^***^p < 0.001, ^**^p < 0.01, ^*^p < 0.05.

**Table 10:** ***F*-test (type III sums of squares) control experiment (DV: ΔSICI)**.

**Coefficients**	***F*-value (df)**
Intercept	0.05 (1, 764)
TIME POINT	0.00 (1, 764)
STIMULATION DURATION	12.83^***^ (1, 764)
SETTING	7.76^**^ (1, 764)
TIME POINT × STIMULATION DURATION	1.30 (1, 764)
TIME POINT × SETTING	4.13^*^ (1, 764)
STIMULATION DURATION × SETTING	0.45 (1, 764)
TIME POINT × STIMULATION DURATION × SETTING	1.81 (1, 764)

*** p < 0.001,

** p < 0.01,

* p < 0.05.

**Figure 5:**
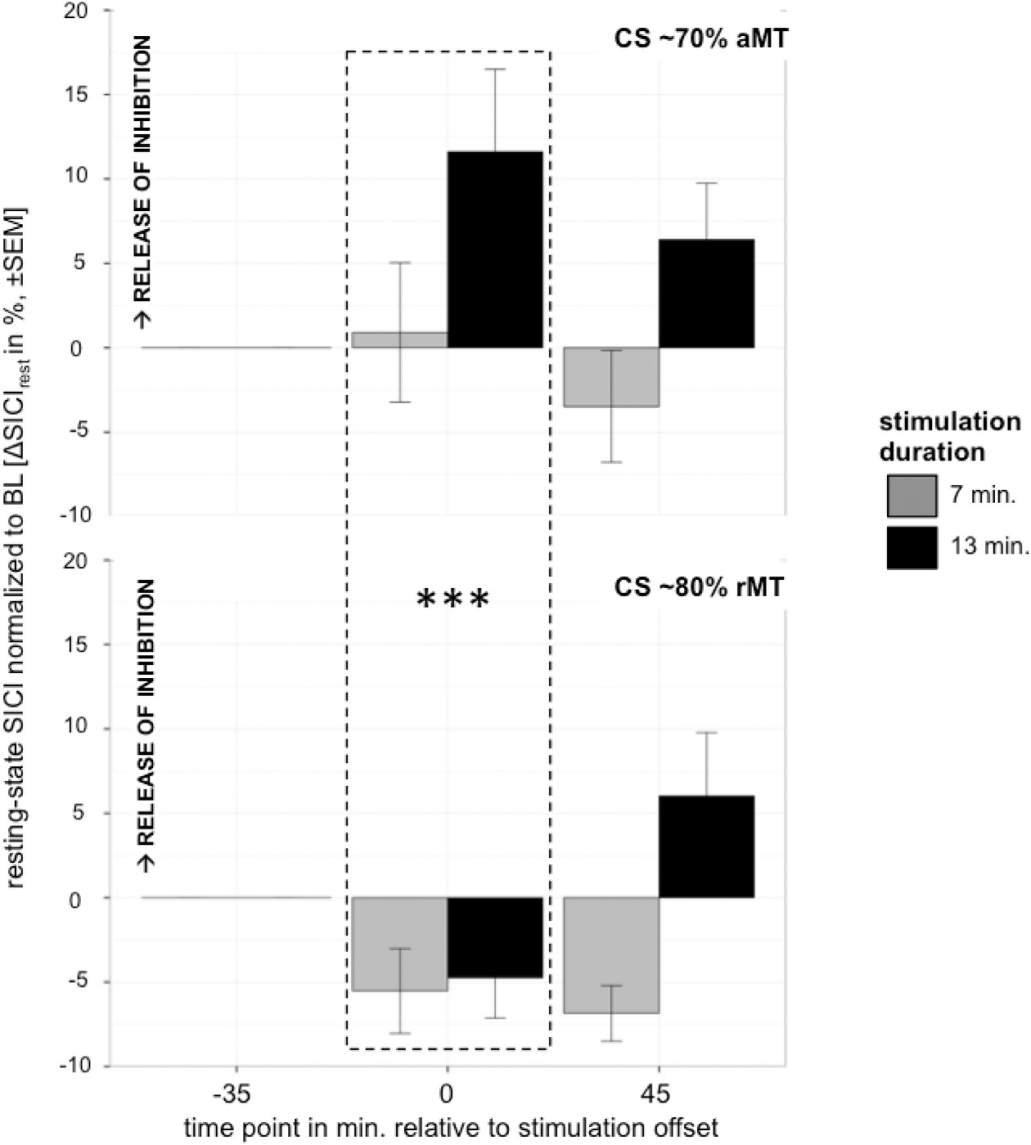
**Control experiment testing effect of different settings with CS intensity variation upper graph: 70%aMT (setting 2), bottom graph: 80%rMT (setting 1) on atDCS effect of 7 min (gray) and 13 min (black) stimulation duration over time**. CS intensity significantly modulated direction of atDCS effect over time. ^***^p < 0.001, ^**^p < 0.01, ^*^p < 0.05, °*p* < 0.1.

A significant SETTING × TIME POINT interaction (*p* < 0.05) showed that the stimulation induced release of inhibition with setting 2 as well as increase in inhibition with setting 1 weakened over time, which was confirmed by *post-hoc* testing (time point P, setting 1 vs. setting 2: 11.47 ± 3.39, *z* = 3.38, *p* < 0.005; time point P45, setting 1 vs. setting 2: 1.65 ± 3.40, *z* = 0.49, *p* > 0.9), i.e., no difference between SICI measured with setting 1 or setting 2 was observable at 45 min after stimulation cessation. Time after stimulation cessation did not modulate the effect of duration of stimulation (Figure 5).

## Discussion

In the present data five main results were found: firstly and most strikingly, atDCS led to differential effects on resting-state inhibition (SICI_rest_) in younger and older participants. While a relative release of inhibition was found in the young, the opposite, a relative increase in inhibition was found in the older. Despite its direction, the atDCS effect also tended to be distinct among age groups with regard to extent and timing. The relative increase in inhibition in the older was evident immediately after stimulation cessation and remained at this level until 90 min following stimulation offset. In the younger however, level of resting-state inhibition did not change significantly from baseline until 90 min after stimulation cessation.

Secondly, stimulation-induced change in event-related modulation of inhibition (SICI_move_) was merely observable in the older who typically demonstrate lost capacity to release inhibition during movement preparation. This was shown by a tendency toward increased modulatory capacity after atDCS in the older subgroup.

Thirdly, initial modulatory capacity, i.e., modulation of event-related inhibition prior to stimulation, was a predictor for the direction and magnitude of the atDCS-induced effects in resting-state inhibition. Specifically older participants with more pronounced modulation of event-related inhibition, i.e., preserved (“young”) modulatory capacity, showed larger atDCS impact in terms of a relative release of resting-state inhibition resembling the disinhibitory effect observed in the younger.

Fourthly, with regard to the behavioral findings, dexterous manual performance was in general positively influenced by atDCS. In accordance with previous findings, the extent of this effect varied with the nature of the task performed (task-specificity) and the beneficial effect was target-group specific, i.e., it was mainly seen in the older. And finally, in terms of associations between net changes in physiology and behavior, a more incongruent picture was found. For 1FT and 2FT, in general less pronounced net resting-state inhibition was associated with comparatively faster performance. In contrast, net release of resting-state inhibition tended to correlate with performance decline in CRT.

### Increase of event-related SICI modulation in the older

In accordance with previous findings (Heise et al., 2013), fast and precise event-related modulation of SICI in terms of a rapid release of inhibition usually observed before movement onset (Reynolds and Ashby, 1999; Gilio et al., 2003; Sinclair and Hammond, 2008), was weakened or even absent in older participants.

Strikingly, atDCS had no effect on the modulation of event-related inhibition in the younger. But event-related modulation tended to increase after atDCS in the older participants. No association however, was found between stimulation-induced changes in resting-state and event-related inhibition. On the one hand, event-related modulation of inhibition (SICI_move_) might reflect at least in parts different processes than resting-state measurements of inhibition (SICI_rest_). On the other hand, event-related modulation of intracortical inhibition in terms of a rapid release of inhibition toward movement onset seems to follow a fairly hard-coded process as long as the underlying mechanisms are intact. Although atDCS weakened resting-state inhibition levels in the younger, event-related release of inhibition was not compromised. In the older age group on the contrary, event-related modulation tended to increase with atDCS. This finding clearly contrasts the initial expectation regarding the atDCS effect, based on the assumption that event-related SICI modulation depends on resting-state levels of inhibition (Heise et al., 2013). It might rather be that initial event-related modulation indicates and predicts overall modulatory capacity of the motor system, and hence the extent to which perturbation is generally possible.

These findings stress the hypothesis that atDCS effects are highly dependent on the state of the cortical network stimulated, as implied by findings of a differentiation of effects among tasks (Boggio et al., 2010; Hummel et al., 2010; Bullard et al., 2011; Leite et al., 2011, 2013) or target groups (Hummel et al., 2010; Tseng et al., 2012; Zimerman et al., 2013) or phase within plastic changes as for example in learning and memory consolidation (Dockery et al., 2009; Kantak et al., 2012; Saucedo Marquez et al., 2013).

### Direction and latency of the atDCS effect on resting-state SICI

So far, the available data in younger (Nitsche et al., 2004, 2005; Kidgell et al., 2013) and one study in older participants (Goodwill et al., 2013) reported a disinhibitory effect (release of inhibition) after a single session of atDCS when applied during resting-state. The present observation of a relative increase of inhibition in the older subgroup is therefore somewhat surprising and in disagreement with existing theories about the mode of action of atDCS at first sight. On the basis of experiments using dpTMS or neurotransmitter-specific MRS, a potential involvement of GABAergic mechanisms has been suggested for atDCS effects, leading to a weakened influence of GABAergic inhibition in young healthy volunteers (Nitsche et al., 2004, 2005; Stagg et al., 2009, 2011b). Based on pharmacological evidence and electrophysiological observation of the temporal pattern, SICI at 3 ms ISI is assumed to represent phasic inhibitory mechanisms, which are synaptically mediated at the GABA_A_ receptor (Davies et al., 1990; Di Lazzaro et al., 1998, 2006; Ilic et al., 2002) but also with certain influence of GABA_B_-ergic activity (Ziemann et al., 1996a; Werhahn et al., 1999; Sanger et al., 2001). Assuming an imbalance of excitatory and inhibitory neuronal activity in the older, findings of weakened short-latency intracortical facilitation (SICF, measured with ISIs of 2.5 ms) in older individuals (Clark et al., 2011) might explain the lack of disinhibition after atDCS observed in the present older group. In consideration of a potential excitatory influence of atDCS on neuronal populations mediating SICF, this effect may be diminished in the older. On the other hand, *in vitro* experiments and first pharmacological studies suggest a calcium-dependence of atDCS after-effects, i.e., atDCS have been shown to increase intracellular Ca^2+^ levels (Nitsche et al., 2003a; Khatib et al., 2004; Dube et al., 2012). In a recent study, a conversion of the after-effect of atDCS on corticospinal excitability was found with prolonged stimulation, which was not observed under the influence of a calcium channel blocker (flunarizine) (Monte-Silva et al., 2013). The question is, how atDCS influences a system, which potentially presents with already elevated intracellular Ca^2+^ levels as observed during the process of healthy aging (Thibault et al., 2001; Murphy et al., 2004). The “calcium-dysregulation hypothesis of brain aging” has been formulated in the late 1980s and has been supported since then, e.g., by calcium imaging of hippocampal neurons (Gibson and Peterson, 1987; Landfield, 1987; Disterhoft et al., 1994; Mattson, 2007). In line with this hypothesis, very recent work has shown increased intracellular calcium buffer in aged rat hippocampal neurons, which was interpreted to represent a focused homeostatic mechanism counteracting elevated intracellular calcium levels (Oh et al., 2013). Therefore, the observed increase of intracortical inhibition in the present older subgroup might indicate a rebound mechanism to prevent over-excitation (Monte-Silva et al., 2013).

Although the expected disinhibitory effect was found in the younger subgroup indeed, the timing of the observed effect clearly contrasts that of the published findings since release of inhibition was not observable immediately after stimulation cessation. To evaluate whether methodological differences might explain this differences, a control experiment was conducted. This analysis revealed a significant interaction of the dpTMS paradigm with the time after stimulation cessation in terms of direction and temporal pattern of SICI induction. This means lower CS intensity resulted in more pronounced release of inhibition (disinhibition) earlier after stimulation.

The clear and immediate disinhibition after atDCS observed in earlier work might well be attributed to excitability shifts within the excitatory interneuronal population rather than modulation of GABAergic inhibitory interneuronal activity. Kujirai's original work suggested most pronounced inhibition with CS intensities of 70%rMT or 90%aMT, above which less SICI induction was explained by possible facilitatory processes superimposing inhibition (Kujirai et al., 1993). Extensive analyzes of the effects of stimulus intensity variations and their interactions with interstimulus intervals have shown less induction of inhibition at SICI protocols using around 3 ms ISI and intensities as low as 70%aMT (Peurala et al., 2008), representing the parameters tested in setting 2 in the present control experiment. However, these data have shown stable inhibition at 90%aMT approximately corresponding to 80%rMT (Chen et al., 1998) as used in the present work. In line with these findings, average baseline SICI of about 40% (i.e., 60% reduction) was found in the present younger subgroup. This resembles and even exceeds 50–80% SICI (i.e., 20–50% reduction) observed in experiments using CS intensities of 70%aMT (Nitsche et al., 2004, 2005; Kidgell et al., 2013). SICI (at 3 ms ISI) measured with lower CS intensities of around 70%aMT has been suggested to represent higher contamination with facilitatory effects, such as SICF (Peurala et al., 2008), which is thought to reflect the activity of excitatory interneuronal interaction contributing to the I-wave generation (Hanajima et al., 2002; Ilic et al., 2002).

Future work needs to examine in detail the potential influence of transcranial direct current stimulation on the recruitment of SICI in participants of older age to advance the understanding of the underlying mechanisms.

### Differential effects on behavior

Previous data has shown improvements of manual motor performance in terms of speed (Boggio et al., 2006; Vines et al., 2006, 2008; Hummel et al., 2010), endurance of muscle force (Cogiamanian et al., 2007), or precision (Matsuo et al., 2011) after atDCS over MI contralateral to the active hand. Here, the effect of atDCS on motor behavior differed among age groups in extent and timing and was dependent on the character of the task performed. While 1FT performance generally declined with continuing tapping over time, performance levels remained stable in the older group after atDCS. Also in CRT, atDCS was only beneficial for the performance in the older group and did not impact on the younger group's performance. In contrast, the complex dexterous coordination of alternating tapping of two different effectors tested with 2FT clearly improved in the atDCS condition compared to sham. While the older group showed a clear performance benefit under atDCS, performance of the younger seemed not to be influenced by the stimulation. Previous data however, support the notion that the atDCS effect is less pronounced when the motor system is optimally tuned, e.g., in the dominant in contrast to the non-dominant hand (Boggio et al., 2006; Vines et al., 2008) or in younger in contrast to older participants (Hummel et al., 2010). It might well be, that atDCS-induced performance gains would have been more pronounced in the younger group if a more challenging task would have been used or the non-dominant hand/hemisphere had been the target area in the present experiments.

Furthermore, a growing body of data shows the specificity of atDCS effects with respect to the task performed for a variety of cognitive and motor domains (Boggio et al., 2010; Hummel et al., 2010; Bullard et al., 2011; Leite et al., 2011, 2013; Meinzer et al., 2012). The present finding of performance increases under atDCS in the more complex tasks is in accordance with previous results showing a distinction of stimulation effects with respect to the nature of the respective task performed. Greater improvements were found in tasks requiring a higher level of dexterity compared to more gross motor function tasks (Hummel et al., 2010). Only few previous studies have analyzed the potential association between atDCS-related changes in parameters of corticospinal excitability and behavioral changes. These studies imply a causal relationship between better performance and increased levels of excitability (Hummel et al., 2005; Stagg et al., 2009; Zimerman et al., 2012).

In the current data however, no correlation was found between stimulation-induced changes to SICI_move_ and behavior. The fairly disparate picture of the correlations between stimulation-induced changes in SICI_rest_ and change in 2FT or CRT performance makes the interpretation difficult, hence speaking against a simple linear relationship of changes in GABA_A_-mediated motorcortical inhibition with performance changes as measured in the present experiments.

In the case of 1FT performance, slowing of isolated finger tapping has been described before and suggested to represent changes in motor control rather than muscle fatigue, i.e., indicating the transition from an “alternating flexion/extension muscle pattern to a less effective co-contraction pattern” with continuing performance (Rodrigues et al., 2009). One explanation for the observed stability in the performance level of 1FT under atDCS in the older subgroup might be the preserved selectivity of motor control with increased intracortical inhibition, which might have been hampered by elevated disinhibition as seen in the younger.

Like Goodwill and colleagues the present work also exposed improvements in the sham condition for the most difficult 2FT task, hence skill acquisition after the initial familiarization phase has to be assumed. The secondary analysis of the improvement in the 2FT task revealed an overall improved skill level, i.e., learning curve (linear slope), under atDCS compared to the sham condition in both age groups. Therefore, it is conceivable that atDCS modulates the within session 2FT learning curve (quadratic and cubic trend) leading to faster improvements early in the practice session in the older age subgroup, while later practice gains were boosted in the younger subgroup. In the older, large performance changes in the beginning of the experiment could indicate an extended time span needed to familiarize with the task or the timing-specific influence of atDCS effects early in motor practice. In younger individuals, performance might have reached already a more stable level, or ceiling, early on. As for the late performance gains in the younger, a possible explanation could be first off-line effects of consolidation after the 45 min intersection between test blocks—an effect, shown to be compromised in older individuals (Brown et al., 2009; Zimerman et al., 2013).

A moderate association between steeper learning curves and the reduction of modulatory capacity was observed in the younger under atDCS. On a speculative note, one might assume a plasticity-inducing process due to skill acquisition during repeated 2FT performance, which has been shown to coincide with a reduction of GABAergic motorcortical inhibition (Bütefisch et al., 2000; Ziemann et al., 2001; Floyer-Lea et al., 2006; Stagg et al., 2011a), adding to the disinhibitory effect of atDCS. Therefore, this might be interpreted as an indicator of immediate physiological homeostatic mechanisms, which limit the induction of plasticity to stabilize the excitation/inhibition balance and maintain network integrity.

### Limitations

Inter-individual variability of (physiological and behavioral) effects of tDCS is a frequently reported observation (Datta et al., 2012; Saucedo Marquez et al., 2013; Wiethoff et al., 2014) and was also seen in the present study. Recent work using threshold-tracking techniques to quantify SICI proposed differential effects of tDCS on early and late components of SICI (Cengiz et al., 2013), i.e., reduction of late SICI and enhancement of early SICI. The authors furthermore suggested individual variability in the exact timing of early and late components and some degree of overlapping rather than fixed intervals. It might therefore be, that differences in the current findings are related to more pronounced influence of these different elements of SICI. As seen in the younger sample who participated in the control experiment, it is conceivable, that methodological adjustments of direct current stimulation and SICI measurement protocols impact differently in the older.

Recent results do not unambiguously support the notion that the effect of tDCS might partly be ascribed to the effect of simultaneous stimulation of motor association cortices covered by large electrodes and their respective input to MI (Kirimoto et al., 2011; Lindenberg et al., 2013). Utilizing a small electrode surface of the active electrode (3.5 m^2^ active electrode, 35 m^2^ reference) to focus current flow to respective intrinsic hand muscle representation, Boros and colleagues did not observe any effect of atDCS over MI on SICI in their control experiment while they observed a significant reduction of inhibition with atDCS to the premotor cortex (Boros et al., 2008). The authors argue that previous findings of their group of SICI reduction after MI stimulation with large electrodes could be attributed to the effect of simultaneous premotor stimulation covered by 35 m^2^ electrodes. Nonetheless, with an electrode size of 25 m^2^ centered at the FDI representation in MI as used in the present experiments, parallel stimulation of adjacent premotor areas cannot be completely ruled out. It might also be of relevance that electrical field strength is not necessarily highest in the cortical regions directly underlying the electrodes, as estimated from MR-derived computational head models (Miranda et al., 2013). It cannot be excluded that performance changes are to a lesser extent related to intracortical mechanisms than to stimulation-induced changes within a broader network. Recent analyzes of uni- and bilateral tDCS with resting-state fMRI have provided evidence for changes in intra- and interhemispheric functional connectivity within primary and secondary motor areas (Lindenberg et al., 2013; Sehm et al., 2013).

It has been suggested that muscle fatigue as well as cognitive strain during the stimulation might interfere with and diminish the effect of atDCS in contrast to stimulation applied in the absence of any motor or cognitive activity (Antal et al., 2007; Thirugnanasambandam et al., 2011). Different from these earlier results in the present experiment, no isometric force production but phasic muscle function was required. Moreover, MEP amplitudes were not observed to decline with task performance, which has been proposed to occur during muscular fatigue (Brasil-Neto et al., 1993; Samii et al., 1996; Sacco et al., 2000; Zijdewind et al., 2000). Nonetheless, it is possible that the cognitive load during the tasks was higher for the older individuals and hence the effect of atDCS might have been comparatively reduced in the older. It cannot be excluded on the other hand, that older individuals were more affected by attention decline or fatigue at the latest time point, although not indicated by self-reported levels on the visual analog scale (supplemental online results).

## Conclusion

Taken together, these findings show that behavioral and physiological effects are age-group dependent with regard to direction, extent, and timing. Initial modulatory capacity potentially serves as a predictor for the responsiveness to an intervention with atDCS. This means that in older the physiologically “younger” the neuronal network in terms of modulatory capacity the more likely does atDCS lead to a transient disinhibitory effect and respective behavioral improvement. These findings strengthen the hypothesis that the underlying mechanisms are dependent on the specific functional state of the motor-cortical network stimulated and hence may lead to disparate effects of stimulation.

It has to be assumed that the relationship between stimulation-induced changes to the GABA_A_ergic system and dexterous manual behavior is not linear but rather influenced by other systems, such as glutamatergic and NMDA-mediated mechanisms. Future work needs to further explore the physiologic mechanism underlying tDCS-induced changes to behavior in order to specifically tailor the application of tDCS to the requirements of the target-groups.

## Author contributions

Kirstin-Friederike Heise conceptualized the study, acquired and analyzed the data, and wrote the manuscript, Martina Niehoff helped with data acquisition and data management, J.-F. Feldheim helped with data management and analysis, Gianpiero Liuzzi helped with conceptualization of the initial study; Christian Gerloff and Friedhelm C. Hummel conceptualized and supervised the study, were involved in data interpretation and writing of the manuscript. All authors have read and approved the final submission.

## Funding

This research was supported by the University of Hamburg (NWF-10/04 to Kirstin-Friederike Heise) and the German Research Foundation (SFB 936-C4 to Friedhelm C. Hummel and SFB 936-C1 to Christian Gerloff).

### Conflict of interest statement

The authors declare that the research was conducted in the absence of any commercial or financial relationships that could be construed as a potential conflict of interest.
