# Supplementary material for: Differential behavioral and physiological effects of anodal transcranial direct current stimulation in healthy adults of younger and older age
Source: Front Aging Neurosci. 2014 Jul 10;6:146. doi: 10.3389/fnagi.2014.00146 (PMC4091308; doi:10.3389/fnagi.2014.00146)
Supplement: Supplementary file 1 [file Presentation1.PDF]

Supplemental Results

Resting state SICI

For analyses of absolute values of dependent variables, natural logarithmic transformation (log) sufficiently assured data distribution in accordance with the central limits theorem.
Model comparison for $SICI_{rest}$ (log) showed a model improvement (i.e. smaller BIC) by adjusting for intra-individual variance by adding unconditioned MEP amplitude to random the random term and allowing for a random intercept of STIMULATION CONDITION to be nested within SUBJECT, showing significant intra-individual variance to be influenced by stimulation condition.
A main effect of AGE GROUP ($F(1, 30)=11.30$, $p<.005$) revealed a marked difference in overall SICI induction between the two age groups. Younger participants presented on average with 0.41 = 41% smaller MEP amplitudes corresponding to -0.89±0.289 on the log scale, i.e. stronger inhibition.
There was a significant main effect of TIME POINT ($F(3, 3842)=10.28$, $p<.0001$), showing a significant change in $SICI_{rest}$ over time relative to BL, which was modulated by age group, TIME POINT x AGE GROUP ($F(3, 3842)=2.87$, $p<.05$), and marginally modulated by stimulation, TIME POINT x STMULATION CONDITION ($F(3, 3842)=2.56$, $p=.05$). Hence, in the next step, change in resting-state SICI ($\Delta SICI_{rest}$) was modelled as DV.

Event-related SICI

Absolute event-related $SICI_{move}$ modulation was drastically diminished in the older group irrespective of stimulation condition or time point of measurement ($F(1, 7147)=20.84$, $p<.001$). A significant intra-individual variation of SICI modulation was confirmed by significant model improvement (smaller BIC) allowing for random slope and intercept. $SICI_{move}$ modulation declined over time (pTIMEST x TIME POINT, $F(3, 7147)= 3.35$, $p<.05$) and this interaction was more pronounced in the younger (TIME POINT x pTIMEST X AGE GROUP, $F(3, 7147)=6.59$, $p<.001$). A significant effect of $SICI_{move}$ modulation (slope) at BL ($F(1, 29)=42.54$, $p<.001$) indicated a reduction of event-related $SICI_{move}$ of 11% corresponding to -2.17±0.33 on the log scale with one unit steeper BL slope. Stimulation did not effect absolute $SICI_{move}$ modulation, i.e. neither main effect nor any of its interactions were significant.

Analysis of 2FT learning curve

Table a1: *Wald statistics learning curve 2FT*

**Coefficients**	**Parameter Estimate (Std. Error)**	**t-value (df)**
(Intercept)	2.79<br>(2.21)	1.26<br>(18557)
AGE GROUP (older)	-0.77	-0.24

	(3.19)	(30)
STIMULATION CONDITION (atDCS)	-0.21<br>(3.08)	-0.07<br>(30)
poly[TIME 3]$_{linear}$	-847.21<br>(76.96)	-11.01***<br>(18557)
poly[TIME 3]$_{quadratic}$	-84.18<br>(5.42)	-15.52***<br>(18557)
poly[TIME 3]$_{cubic}$	81.02<br>(4.17)	19.44***<br>(18557)
AGE GROUP (older) x STIMULATION CONDITION (atDCS)	-0.37<br>(4.45)	-0.08<br>(30)
AGE GROUP (older) x poly[TIME 3]$_{linear}$	392.62<br>(108.84)	3.61***<br>(18557)
AGE GROUP (older) x poly[TIME 3]$_{quadratic}$	15.93<br>(7.67)	2.08*<br>(18557)
AGE GROUP (older) x poly[TIME 3]$_{cubic}$	-59.55<br>(5.90)	-10.10***<br>(18557)
STIMULATION CONDITION (atDCS) x poly[TIME 3]$_{linear}$	67.14<br>(93.60)	0.72<br>(18557)
STIMULATION CONDITION (atDCS) x poly[TIME 3]$_{quadratic}$	3.10<br>(7.67)	0.40<br>(18557)
STIMULATION CONDITION (atDCS) x poly[TIME 3]$_{cubic}$	-19.13<br>(5.90)	-3.24**<br>(18557)
AGE GROUP (older) x STIMULATION CONDITION (atDCS) x poly[TIME 3]]$_{linear}$	-10.79<br>(132.37)	-0.08<br>(18557)
AGE GROUP (older) x STIMULATION CONDITION (atDCS) x poly[TIME 3]$_{quadratic}$	34.62<br>(10.85)	3.19**<br>(18557)
AGE GROUP (older) x STIMULATION CONDITION (atDCS) x poly[TIME 3]$_{cubic}$	23.02<br>(8.34)	2.76**<br>(18557)
**Random effects**	**Variance (Std.Dev)**	**Correlation**
**~time|ID**		
(Intercept)	4.30^{-11} (6.56^{-06})	(Intercept)
time	6.0^{-04} (2.45^{-02})	0.68
**~time|ID/STIMULATION CONDITION**		
(Intercept)	4.49^{-20} (2.12^{-10})	(Intercept)
time	1.66^{-03} (4.08^{-02})	0.42
Residual	8.53^{+01} (9.24)	
**Correlation Structure AR(1)**	**Formula: ~1|ID**	
Parameter estimates: Phi	0.99	

**** p < 0.001, ** p < 0.01, * p < 0.05, °p<.1*

Results of VISUAL ANALOGUE SCALE (VAS) estimation of self-perceived level of attention and fatigue.

Linear regression with time point (integer), group (older, younger), and stimulation condition (sham, atDCS) added to the model as main effects and interactions for independent variables and with VAS score (0 maximal attentive/awake – 10 maximal inattentive/tired) for either attention or fatigue as dependent variable.

Self-estimated level of attention

Residual standard error: 1.6 on DF=248

Multiple R^2=.0683, Adjusted R^2= .042
F(7, 248)=2.6, p< .05

Table a2:

**Coefficients:**	**Estimate ± SEM**	**t value**
(Intercept)	2.40±0.23	10.60***
TIME POINT	-0.00±0.00	-0.13
AGE GROUP (older)	-0.72±0.32	-2.26*
STIMULATION CONDITION (atDCS)	-0.00±0.32	0.00
TIME POINT x AGE GROUP (older)	0.00±0.01	0.62
TIME POINT x STIMULATION CONDITION (atDCS)	0.00±0.01	1.10
AGE GROUP (older) x STIMULATION CONDITION (atDCS)	-0.11±0.45	-0.23
TIME POINT x AGE GROUP (older) x STIMULATION CONDITION (atDCS)	-0.00±0.01	-0.50

**** p < 0.001, ** p < 0.01, * p < 0.05, °p<.1*

Self-estimated level of fatigue

Residual standard error: 1.66 on DF=248
Multiple R^2: 0.0861, Adjusted R^2: .06
F(7, 248)= 3.34, p< .005

Table a3:

**Coefficients:**	**Estimate±SEM**	**t value**
(Intercept)	2.80±0.24	11.90***
TIME POINT	0.00±0.04	0.21
AGE GROUP (older)	-1.16±0.33	-3.49***
STIMULATION CONDITION (atDCS)	-0.14±0.33	-0.43
TIME POINT x AGE GROUP (older)	0.00±0.01	0.33
TIME POINT x STIMULATION CONDITION (atDCS)	0.00±0.06	0.05
AGE GROUP (older) x STIMULATION CONDITION (atDCS)	0.28±0.4.7	0.60
TIME POINT x AGE GROUP (older) x STIMULATION CONDITION (atDCS)	0.00±0.01	0.00

******* *p < 0.001, ** p < 0.01, * p < 0.05, °p<.1*

Supplemental Figures

additional Figure a1

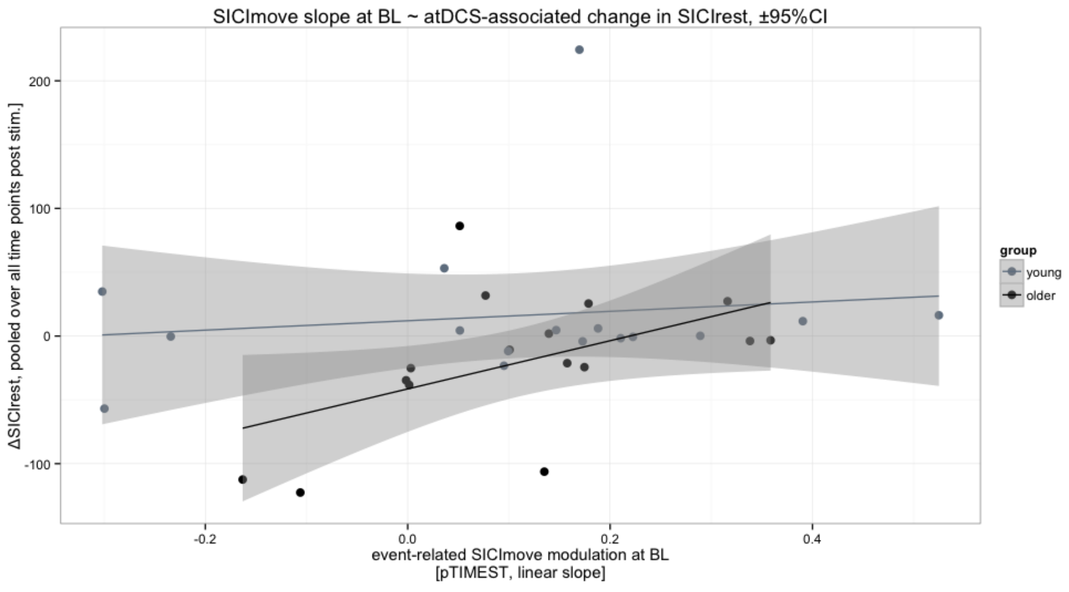

modulatory capacity

Figure a1: More pronounced initial event-related $SICI_{move}$ modulation (modulatory capacity) tended to be associated with larger change to resting-state SICI under atDCS (r_{pb}=.53, CI= -.04 – .87, $T_{pb}(14)$=2.36, p=.06)

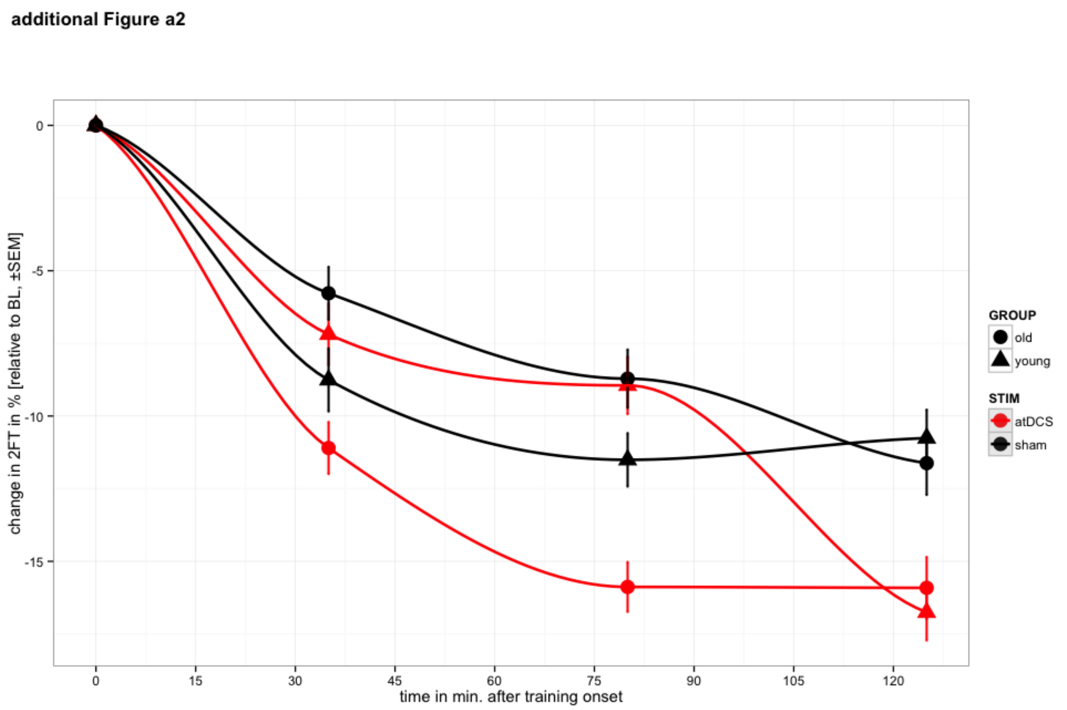

Figure a2: Anodal stimulation modulated the curvilinear temporal pattern of performance improvement in the 2FT task over time, i.e. learning curve, differently in the two age groups ($p<.0005$).
